# Supplementary material for: A PSII photosynthetic control is activated in anoxic cultures of green algae following illumination
Source: Commun Biol. 2023 May 12;6:514. doi: 10.1038/s42003-023-04890-3 (PMC10182038; doi:10.1038/s42003-023-04890-3)
Supplement: Supplementary file 2 — Supplementary figures [file 42003_2023_4890_MOESM2_ESM.pdf]

Supplementary data

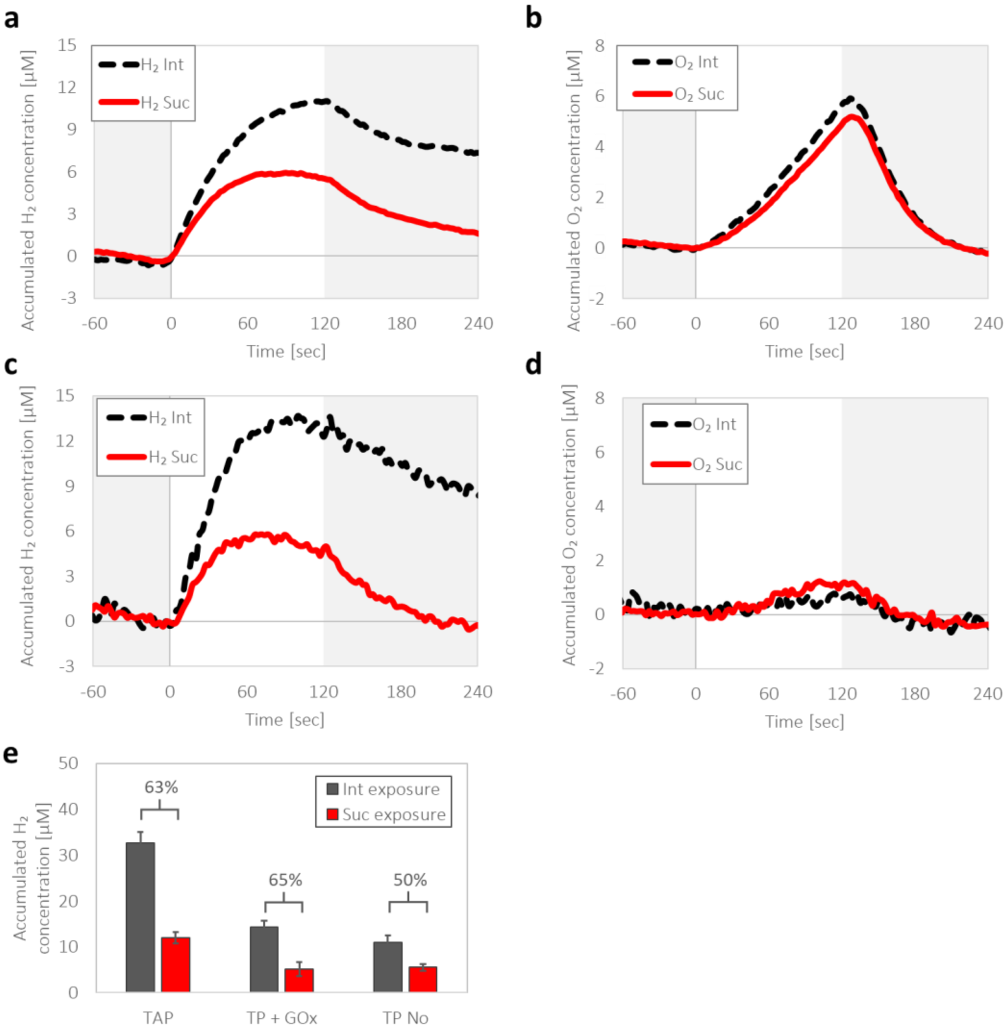

Supplementary Figure 1: Hydrogen and oxygen evolution under autotrophic conditions. *C. reinhardtii* (wild-type strain- CC124) cells were cultivated under autotrophic conditions (TP, pH 7.2, supplied with 5% CO_2) and incubated for an hour under dark anaerobiosis, after which they were challenged with light fluctuations of 2 minutes under illumination (at an irradiance of 370 µE m^{-2} s^{-1}, white background), followed by 3 minutes of darkness (gray background, as shown in Figure 1). The measurements were done in the absence (**a**,**b**) or presence (**c**,**d**) of O_2 scavangers (Gox). Shown are the differences between the initial light exposure (dashed black) and the average of the successive exposures (solid red). H_2 (**a,c**) and O_2 (**b,d**) concentrations were measured. Accumulated H_2 concentration were determined and plotted in respect of each light exposure (**e**, initial exposure in black, and succesive exposures in red). The ratio between exposures also calculated (see presentage for each coupled columns). Graphs represent an average of three biological repeats, error bars in (**e**) were calculated as standart error.

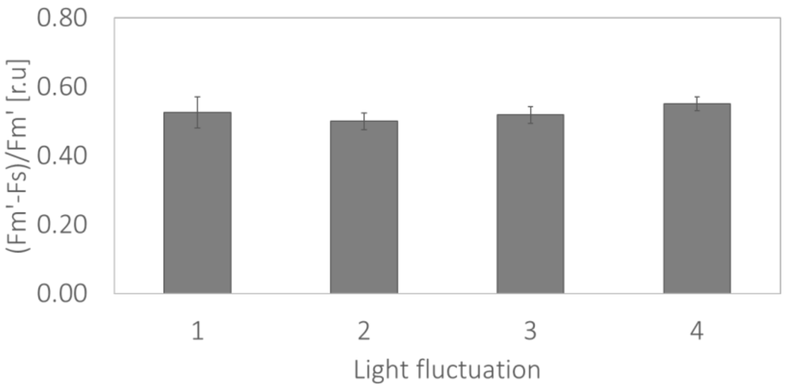

Supplementary Figure 2: Relative maximal fluorescence under light fluctuations. Following an hour of dark anaerobic incubation, *C. reinhardtii* wild-type strain CC124 cells were examined in a Dual-PAM-100 for Chl*a* fluorescence under fluctuating light exposures. During illuminations, the cells were also exposed to saturating light pulses (see yellow arrows in **Figure 1a**) to evaluate the maximal fluorescence (Fm'). Relative quantum yields were calculated as (Fm'-Fs)/Fm' for each fluctuation and compared. Each column represent the averaged result of at least three biological repeats. Error bars represent standard error.

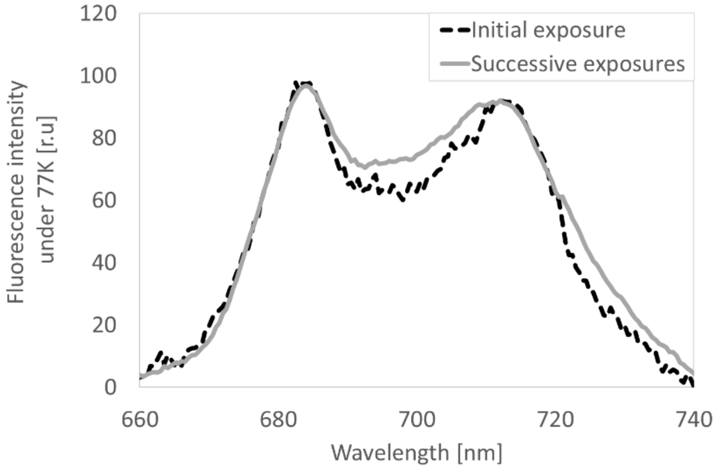

Supplementary Figure 3: Examinations of state transitions during fluctuating light. *C.reinhardtii* wild-type strain CC124 cells were incubated for an hour under dark anaerobiosis, after which they were exposed to 2 minutes of irradiance (370 μE m^{-2} s^{-1}) followed by 3 minutes of darkness. Prior to and in-between illuminations, we sampled the cells and immediately tested their fluorescence spectra under 77K. Shown are the differences between the initial light exposure (dashed) and the average of the successive exposures (solid), relative to the values which were measured at 685nm. Curves represent the averaged results for three biological replicates.

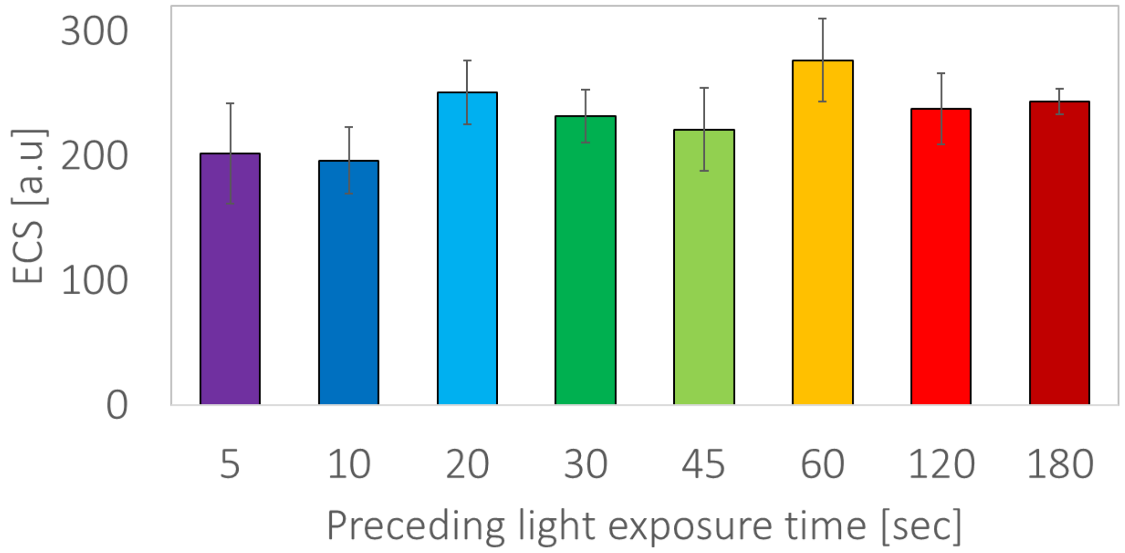

Supplementary Figure 4: Charge separation of laser flashes following illumination. *C. reinhardtii* wild-type strain CC124 cells were incubated for an hour under dark anaerobiosis, after which they tested in JTS-100, equipped with a BiLED (520-546 nm) measuring lamp. The cells were exposed to illumination (at an irradiance of 370 µE m^{-2} s^{-1}) for a duration of either 5, 10, 20, 30, 45, 60, 120 or 180 seconds, after which they were exposed to a 5 ns laser flash. Presented are the values for "phase a" of the charge separation, *i.e* the differences in ECS following less than a millisecond. Columns represent the average of at least three biological repeats. Error bars represent standard error.

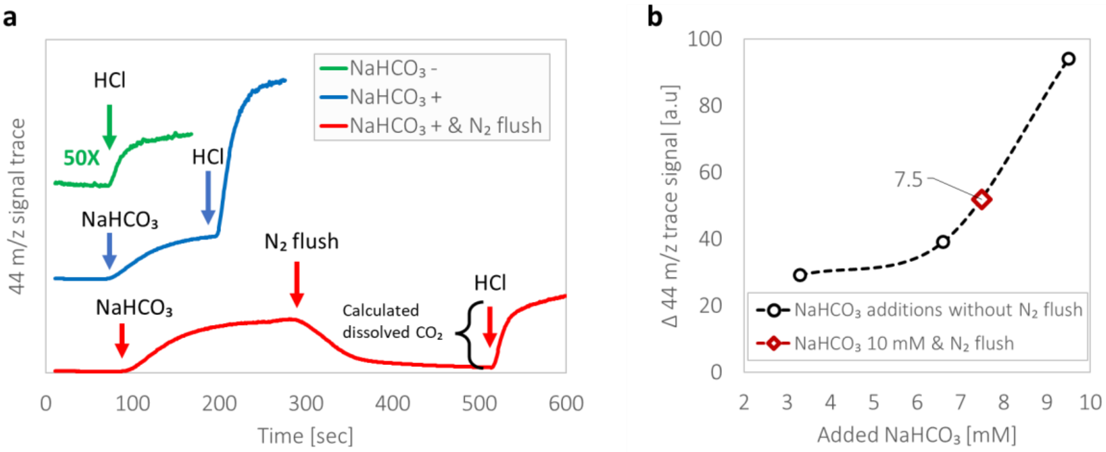

Supplementary Figure. 5: Bicarbonate concentrations following N_2 flush. PSII activity medium (see Materials & Method) was placed in a mambrane inlet mass spectrometer (MIMS), and the signal for 44 m/z (which is correlated to CO_2) was measured *versus* time (**a**). The addition of a strong acid (see arrows, HCl) triggered an immidiate increase in CO_2 due to the shift in pH balance toward its formation. The medium was examined in the absance (green, note that the trace is amplified by a facor of 50X) or following an injection of 10mM $NaHCO_3$ (see arrow, blue). The medium was also measured during a N_2 flush (see arrow, red), afterwhich the acid was added, and the remaining HCO_3^- concentration was determined as the re-evolved CO_2. (**b**) To determine the actual remaining HCO_3^- concentration, the experiment was repeated without N_2 flush with additions of 3.3, 6 and 10 mM $NaHCO_3$ (black- dashed). The amplitude of the re-evolved CO_2 was then ploted to match the fitted concentration and the concentration of the actuall dissolved HCO_3^- was determined to be 7.5 mM (red diamond).

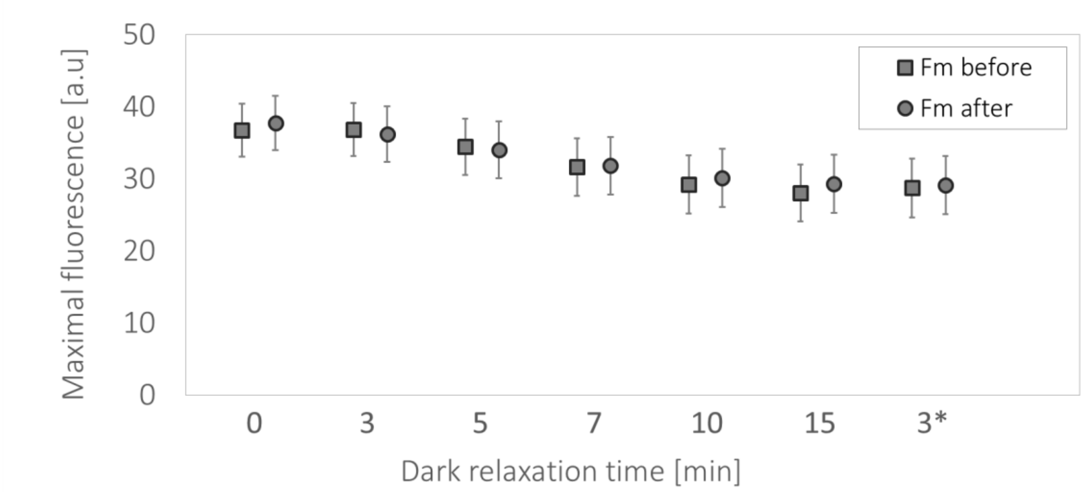

Supplementary Figure. 6: Maximal Chl*a* fluorescence differences as a function of the duration of light exposure. Following an hour of dark incubation in the presence of O_2 scavengers (GOx), *C. reinhardtii* wild-type strain CC124 cells were subjected to a series of light exposures (370 µE m^{-2} s^{-1} for 2 minutes), in which dark relaxation time between exposures was gradually increased (X-Axis). 60 seconds before (square) and 30 seconds after (circle) each illumination, the cells were subjected to a saturating pulse to determine maximal Chl*a* fluorescence (Fm). Error bars indicate standard error ($n \geq 3$).

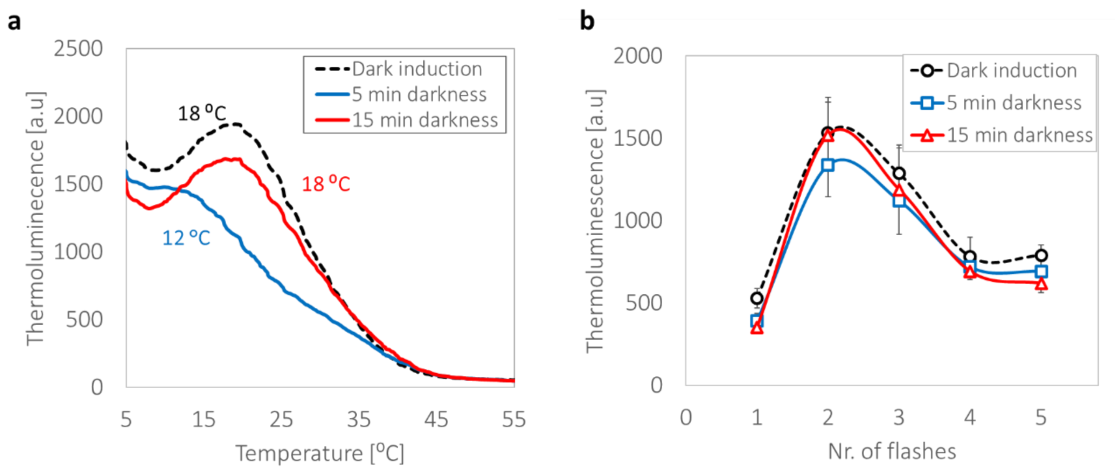

Supplementary Figure. 7: Thermoluminescence results per flash. Cells' thermoluminescence was measured after 1-5 single turnover flashes (STF). Samples were tested following an hour of dark anaerobic incubation (dashed black). They were then illuminated for 2 minutes followed by a dark relaxation of either 5 (blue) or 15 (red) minutes. (**a**) presented are the results for 2 STFs, the temperature of the maximal B-band were determined for further analysis. (**b**) Maximal intensity of the B-band was determined and avaraged for each STF, 2nd flash results in higer signal for all treatements. Each experiment was repeated using three biological replicates, error bars indicate on standart errors.
