# Supplementary material for: A PSII photosynthetic control is activated in anoxic cultures of green algae following illumination
Source: Commun Biol. 2023 May 12;6:514. doi: 10.1038/s42003-023-04890-3 (PMC10182038; doi:10.1038/s42003-023-04890-3)
Supplement: Supplementary file 3 — Description of Additional Supplementary Files [file 42003_2023_4890_MOESM3_ESM.pdf]

Description of Additional Supplementary Files

File name: Supplementary Data 1

Description: An excel file of all the raw data of all the figures in this MS
